# Supplementary material for: Controlling target brain regions by optimal selection of input nodes
Source: PLoS Comput Biol. 2024 Jan 12;20(1):e1011274. doi: 10.1371/journal.pcbi.1011274 (PMC10810536; doi:10.1371/journal.pcbi.1011274)
Supplement: S1 Text — Details on the derivation of the main linear controlability formulas reported in Methods. (PDF) [file pcbi.1011274.s001.pdf]

S1 Text. Linear controllability

A continuous linear time-invariant(LTI) system has a state equation of the form:

$$\dot{\mathbf{x}}(t) = A\mathbf{x}(t) + B\mathbf{u}(t) \quad (1)$$

Here, $\mathbf{x}(t)$ is a $n \times 1$ state vector of the form $(x_1(t), ..., x_n(t))^T$, n being the number of nodes in the system. A is an $n \times n$ matrix describing the interaction strength between the system components. The system is stable if and only if all the eigenvalues of A have negative real part. B is an $n \times r$ input matrix ($r \leq n$) which identifies the nodes controlled by an outside controller, or *driver nodes*, with $B_{ij} = 1$ if control input $u_j(t)$ is imposed on node i; $\mathbf{u}(t)$ is a time-dependent $r \times 1$ input vector of the form $\mathbf{u}(t) = (u_1(t), ..., u_r(t))^T$ with r external inputs.
The system defined by continuous LTI Eq. 8 is said to be *controllable* if for a suitable choice of input signal vector $\mathbf{u}(t), t \in [0, t_f]$, it can be driven from any initial state $\mathbf{x}_0 = x(0)$ to any final state $\mathbf{x}_f = \mathbf{x}(t_f)$, where t_f is a finite time. Kalman's controllability rank condition states that a system is controllable if and only if the *controllability matrix*

$$\mathcal{C} = [B, AB, A^2B, ..., A^{n-1}B] \quad (2)$$

which is of dimension $N \times (N \cdot r)$, has full rank [1]:

$$rank(\mathcal{C}) = n \quad (3)$$

An equivalent condition can be formulated in terms of the *controllability Gramian* W, implicitly defined by the (continuous) Lyapunov equation

$$AW + WA^T = -BB^T \quad (4)$$

Eq. (4) has unique solution

$$W = \int_0^\infty e^{At}BB^Te^{A^Tt} \quad (5)$$

Then the system is controllable if and only if W is positive definite,

$$W > 0 \quad (6)$$

Here, following standard notation, $W > 0$ means $\min(eig(W)) > 0$), i.e., the minimum eigenvalue of W is strictly positive. The conditions (3) and (6) are algebraically equivalent. The two conditions can be numerically verified by computing the minimum singular values of $\mathcal{C}$ and W, respectively. Note that, due to numerical inaccuracies, it is impossible to assess whether a singular value is exactly 0. Therefore, following common practice [2], we consider an eigenvalue to be 0 whenever is it below a very low numerical threshold $\epsilon = 10^{-14}$ (note that fixing a threshold can lead to seeming inconsistencies between the two conditions whenever the minimum eigenvalues are small) [3, 4].

The *control energy* is defined as the (integrated) amplitude of the control signal used to steer the system from a given initial state $\mathbf{x}_0$ to a given final state $\mathbf{x}_f$,

$$E(\mathbf{u}) = \int_0^{t_f} dt||\mathbf{u}(t)||^2 \quad (7)$$

Among infinitely many solutions for $\mathbf{u}$ which can drive the system $\mathbf{x}_0$ to final state $\mathbf{x}_f$ in time t_f, the optimal control input

$$\mathbf{u}^*(\mathbf{x}_0, \mathbf{x}_f, t_f) = B^T e^{A^T(t_f - t)} W(t_f)^{-1}(\mathbf{x}_f - e^{At_f}\mathbf{x}_0)$$

minimizes the control energy [5], i.e.,

$$\begin{aligned} min_{\{\mathbf{u},\ x(t_f)=x_f\}} E(\mathbf{u}) = E(\mathbf{u}^*(\mathbf{x}_0, \mathbf{x}_f, t_f)) = \\ = (\mathbf{x}_f - e^{At_f}\mathbf{x}_0)^T W(t_f)^{-1}(\mathbf{x}_f - e^{At_f}\mathbf{x}_0) = (\mathbf{x}_f)^T W(t_f)^{-1}\mathbf{x}_f \end{aligned} \tag{8}$$

where $W(t_f) = \int_0^{t_f} dt e^{At} B B^T e^{A^T t}$ and we assumed that the final state is normalized, $||\mathbf{x}_f||_2^2 = 1$. The energy cost can be thus bounded as follows:

$$\frac{1}{\lambda_{max}(W(t_f))} \equiv E_{min}(t_f) \leq E(\mathbf{u}^*(\mathbf{x}_0, \mathbf{x}_f, t_f)) \leq E_{max}(t_f) \equiv \frac{1}{\lambda_{min}(W(t_f))} \tag{9}$$

Since for a stable system the real parts of the eigenvalues of A are negative, the optimal control energy $E(\mathbf{u}^*(\mathbf{x}_0, \mathbf{x}_f, t_f))$ quickly decays to a (nonzero) asymptotic value $E^*(\mathbf{x}_0, \mathbf{x}_f) \equiv \lim_{t_f \to \infty} E(\mathbf{u}^*(\mathbf{x}_0, \mathbf{x}_f, t_f))$. In this limit, $W(t_f)$ coincides with the controllability Gramian, $\lim_{t_f \to \infty} W(t_f) = W$ [6]. Hence, the bounds on the energy given by Eq. 9 can be expressed as

$$\frac{1}{\lambda_{max}(W)} \equiv E_{min} \leq E^*(\mathbf{x}_0, \mathbf{x}_f) \leq E_{max} \equiv \frac{1}{\lambda_{min}(W)} \tag{10}$$

The typical metric to assess the difficulty of steering the system from one state to another is given by the upper bound of Eq. 11. This upper bound gives us control energy cost required to steer the brain system to worst possible eigen direction of controllability gramian W and henceforth we consider this quantity as control energy.

$$\mathcal{E} = 1/\lambda_{min}(W) \tag{11}$$

where the λs are simply the eigenvalues of W.

In *target control*, one aims to control only a selected subset of target nodes [7]. The framework is the same, with the only difference that we focus on the final state of a subset of nodes $\mathbf{y}$:

$$\dot{\mathbf{x}}(t) = A\mathbf{x}(t) + B\mathbf{u}(t) \tag{12}$$

$$\mathbf{y}(t) = C\mathbf{x}(t) \tag{13}$$

where $\mathbf{y} \in \mathbb{R}^S$ is the output vector describing the activity of the the target nodes we want to control. Given a network with n nodes, we can define a target node set $\mathcal{T} = \{\tau_1, \tau_2, \tau_3 ... \tau_S\}$ of size $S = |\mathcal{T}|$, where τ_i can be any node from $\{1, 2, 3..., n\}$. The output matrix C is defined as: $C = [\mathbb{I}_{\tau_1}^T, \mathbb{I}_{\tau_2}^T, ..., \mathbb{I}_{\tau_S}^T]^T \in \mathbb{R}^{S \times N}$ where $\mathbb{I}_{\tau_i}$ is τ_i-th row of the identity matrix $\mathbb{I}$.

The definition of target controllability follows from that of standard (Kalman) controllability, where the system is now defined by the triple (A, B, C) instead of the pair (A, B) [7]. The system (A, B, C) is said to be *target controllable* with respect to target node set $\mathcal{C}$ if there exists a time-dependent input vector $\mathbf{u}$ which can drive the state of the target nodes to any desired final state in finite time. It can be shown that the system is target controllable if and only if

$$rank[\mathcal{C}] = S \tag{14}$$

where $\mathcal{C} \equiv [CB, CAB, CA^2B, ..., CA^{n-1}B]$ is the target controllability matrix. The Gramian for target controllability is given by [8]:

$$W_C = CWC^T \tag{15}$$

where W is the standard Gramian, Eq. (9) for system (A, B). Analogously to the case of full controllability, the optimal control input [7]

$$\mathbf{u}_t^* = B^T e^{A^T(t_f - t)} C^T \left(CW(t_f)C^T\right)^{-1} \left(\mathbf{y}_f - CA^{t_f}\mathbf{x}_0\right)$$

which minimizes the control energy and drives the subsystem from initial state $\mathbf{y}_0 = C\mathbf{x}_0$ to final output state $\mathbf{y}_f$ is substituted into Eq. (10) and we obtain the optimal target control energy:

$$E(\mathbf{u}^*(\mathbf{y}_f, \mathbf{y}_0, t_f) = \mathbf{y}_f^T (CW(t_f)C^T)^{-1} \mathbf{y}_f \tag{16}$$

where we have assumed that the initial state is $\mathbf{x}_0 = \mathbf{0}$. The energy can be bounded as follows:

$$\frac{1}{\lambda_{max}(CW(t_f)C^T)} \equiv E_{min}(t_f) \leq E(\mathbf{u}^*(\mathbf{y}_f, \mathbf{y}_0, t_f) \leq E_{max}(t_f) \equiv \frac{1}{\lambda_{min}(CW_{t_f}C^T)}$$

Asymptotically, as $t_f \to \infty$, we can rewrite the above bounds as:

$$\frac{1}{\lambda_{max}(W_C)} \leq E^*(\mathbf{y}_f, \mathbf{y}_0) \leq \frac{1}{\lambda_{min}(W_C)} \tag{17}$$

The upper bound gives the energy required to steer the subsystem along the worst possible direction:

$$\mathcal{E}^{target} = 1/\lambda_{min}(W_C) \tag{18}$$

If a single driver node i is used, and the target is in turn a single node j, the expression of the control energy significantly simplifies. We have $B = \mathbf{e}_i$ and $C = \mathbf{e}_j^T$. Thus

$$\mathcal{E}_{i\to j} \equiv E_{min}^{target} = (W_{jj}^{(i)})^{-1} = \frac{1}{\int_0^\infty dt[e^{At}]_{ji}^2} \tag{19}$$

References

1. Sontag ED. Mathematical control theory: deterministic finite dimensional systems. vol. 6. Springer Science & Business Media; 2013.

2. Yuan Z, Zhao C, Di Z, Wang WX, Lai YC. Exact controllability of complex networks. Nature Communications. 2013;4(1). doi:10.1038/ncomms3447.

3. Tu C, Rocha RP, Corbetta M, Zampieri S, Zorzi M, Suweis S. Warnings and caveats in brain controllability. NeuroImage. 2018;176:83–91.

4. Suweis S, Tu C, Rocha RP, Zampieri S, Zorzi M, Corbetta M. Brain controllability: not a slam dunk yet. Neuroimage. 2019;200:552–555.

5. Yan G, Ren J, Lai YC, Lai CH, Li B. Controlling Complex Networks: How Much Energy Is Needed? Phys Rev Lett. 2012;108:218703. doi:10.1103/PhysRevLett.108.218703.

6. Yan G, Tsekenis G, Barzel B, Slotine JJ, Liu YY, Barabási AL. Spectrum of controlling and observing complex networks. Nature Physics. 2015;11(9):779–786. doi:10.1038/nphys3422.

7. Gao J, Liu YY, D'Souza RM, Barabási AL. Target control of complex networks. Nature Communications. 2014;5(1). doi:10.1038/ncomms6415.

8. Duan G, Li A, Meng T, Wang L. Energy cost for target control of complex networks. Advances in Complex Systems. 2019;22(07n08):1950022.
