# Supplementary material for: Controlling target brain regions by optimal selection of input nodes
Source: PLoS Comput Biol. 2024 Jan 12;20(1):e1011274. doi: 10.1371/journal.pcbi.1011274 (PMC10810536; doi:10.1371/journal.pcbi.1011274)
Supplement: S2 Text — Details optimal nodes to control each target RSN. (PDF) [file pcbi.1011274.s002.pdf]

S2 Text. Optimal nodes to control RSNs according to rank aggregation

For each target RSN, we have ranked nodes according to the centrality measure yielding the lowest average energy, when driver nodes are selected among the nodes with largest centrality. After rankings were estimated separately for each subject, we have used rank aggregation (average rank) to obtain a group ranking. Here, we focus on the 10 top-ranking nodes for each target RSN (Fig. 13). The top nodes to control the CON are located in ventrolateral prefrontal cortex (DMN and VAN), precuneus and right parietal (DMN), frontal eye fields (DAN), right dorsal frontal (DMN). The top nodes to control the DMN are located in left cerebellum, right parietal (DAN), left parietal (CON), frontal eye fields (DAN), ventrolateral PFC (VAN), dorsomedial prfrontal cortex (DMN/VAN). The top nodes to control the DAN are located in right parietal (DMN), right precentral (SMN), left striatum, ventrolateral PFC (VAN/DMN), dorsomedial frontal (VAN), right dorsal frontal (DMN). The top nodes to control the limbic nodes are located in right cerebellum, right temporal cortex (VIS), right precentral (SMN), right frontal eye field (DAN), right dorsal frontal (DMN), ventrolateral prefrontal cortex (DMN and VAN), left dorsolateral preforntal cortex (VAN). The top nodes to control the VAN are located in precuneus (DMN), right parietal (DMN), medial prefrontal cortex (DMN), ventrolateral prefrontal cortex (DMN), frontal eye fields (DAN), right dorsal frontal (DMN and CON). The top nodes to control the SMN are located in striatum, frontal eye fields (DAN), ventrolateral prefrontal cortex (VAN and DMN), dorsomedial frontal (CON), right precentral (DAN). The top nodes to control subcortical regions are located in ventral temporal cortex (limbic), temporal (VIS), ventrolateral prefrontal cortex (DMN and VAN), orbitofrontal cortex (limbic), frontal eye fields (DAN). The top nodes do control the VIS are located in left cerebellum, precuneus (DMN), striatum, right ventral temporal (limbic), ventrolateral prefrontal cortex (DMN/VAN), right frontal eye field (DAN), medial parietal (CON). In general, we observe a large prevalence of anterior nodes, in particular from ventrolateral prefrontal cortex.
