# Supplementary material for: Controlling target brain regions by optimal selection of input nodes
Source: PLoS Comput Biol. 2024 Jan 12;20(1):e1011274. doi: 10.1371/journal.pcbi.1011274 (PMC10810536; doi:10.1371/journal.pcbi.1011274)
Supplement: S1 Fig — The 74 brain areas are divided in left cortical, right cortical and subcortical areas. The figure in panel (C) represents the asymmetry in A, defined as δA = A − AT. (PDF) [file pcbi.1011274.s003.pdf]

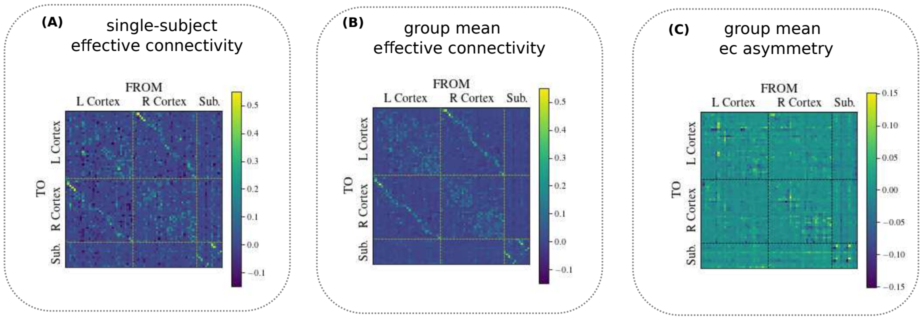

S1 Fig. Examples of effective connectivity matrices. The 74 brain areas are divided in left cortical, right cortical and subcortical areas. The figure in panel (C) represents the asymmetry in A, defined as $\delta A = A - A^T$.
