# Supplementary material for: Controlling target brain regions by optimal selection of input nodes
Source: PLoS Comput Biol. 2024 Jan 12;20(1):e1011274. doi: 10.1371/journal.pcbi.1011274 (PMC10810536; doi:10.1371/journal.pcbi.1011274)
Supplement: S2 Fig — We simulated the propagation of a perturbation in the model x˙(t)=Ax(t) where A is the average (over subjects) effective connectivity matrix. The system’s state was initialized to x(0) = 0 (corresponding to the stable equilibrium point) except for the state of node i that was perturbed to xi(0) = 1. We let the system evolve freely until t = 100. The signal of area j is the functional response of area j to the perturbation in i. (A) functional in all areas j ≠ 0 when node i = 0 is perturbed. (B) For each region (except the pertubed region i), we identified the time corresponding to the maximum of the functional response. We iterated this procedure perturbing all n regions in the network, obtaining n ⋅ (n−1)) response peak times. We show the histogram of peak response times. The average response time is T = 10.29. (PDF) [file pcbi.1011274.s004.pdf]

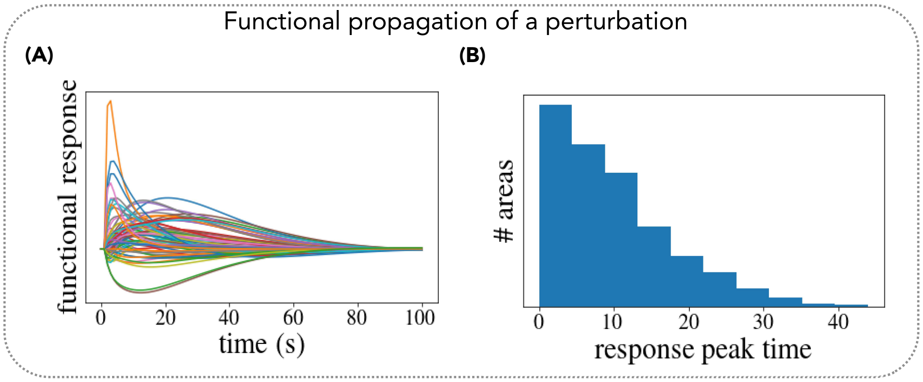

S2 Fig. Functional propagation of perturbations. We simulated the propagation of a perturbation in the model $\dot{\mathbf{x}}(t) = A\mathbf{x}(t)$ where A is the average (over subjects) effective connectivity matrix. The system's state was initialized to $\mathbf{x}(0) = 0$ (corresponding to the stable equilibrium point) except for the state of node i that was perturbed to $x_i(0) = 1$. We let the system evolve freely until $t = 100$. The signal of area j is the functional response of area j to the perturbation in i. **(A)** functional in all areas $j \neq 0$ when node $i = 0$ is perturbed. **(B)** For each region (except the pertubed region i), we identified the time corresponding to the maximum of the functional response. We iterated this procedure perturbing all n regions in the network, obtaining $n \cdot (n-1))$ response peak times. We show the histogram of peak response times. The average response time is $T = 10.29$.
