# Supplementary material for: Controlling target brain regions by optimal selection of input nodes
Source: PLoS Comput Biol. 2024 Jan 12;20(1):e1011274. doi: 10.1371/journal.pcbi.1011274 (PMC10810536; doi:10.1371/journal.pcbi.1011274)
Supplement: S3 Fig — (A) For each subject, we computed the Pearson correlation R between the node driver and target centrality Eid, Eit (average energy to control other nodes from node i vs average energy to control node i from other nodes) and the in-strength Aiin and out-strength Aiout of effective connections, as well as the strength of functional connections Fi. We show the distribution of |R| over subjects. (B) For each node, we computed the coefficient of variation (s.d./mean) over subjects of Eid, Eit, Aiout Aiin. We show the distribution of the coefficient of variation over nodes. (PDF) [file pcbi.1011274.s005.pdf]

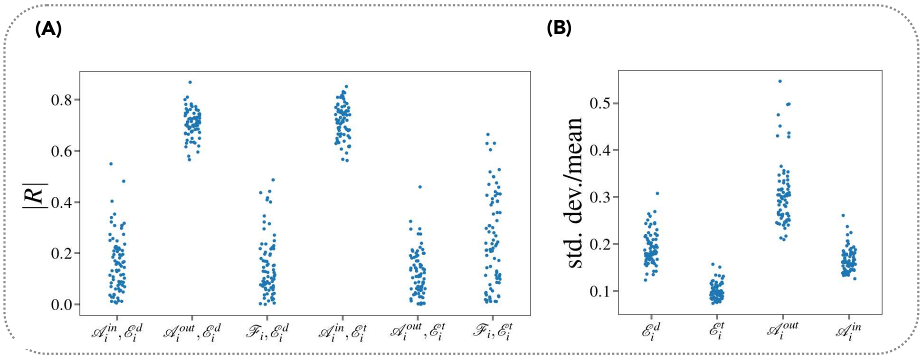

S3 Fig. Relation of driver and target control energy and effective connectivity. **(A)** For each subject, we computed the Pearson correlation R between the node driver and target centrality $\mathcal{E}_i^d$, $\mathcal{E}_i^t$ (average energy to control other nodes from node i vs average energy to control node i from other nodes) and the in-strength A_i^{in} and out-strength A_i^{out} of effective connections, as well as the strength of functional connections $\mathcal{F}_i$. We show the distribution of $|R|$ over subjects. **(B)** For each node, we computed the coefficient of variation (s.d./mean) over subjects of $\mathcal{E}_i^d$,$\mathcal{E}_i^t$, A_i^{out} A_i^{in}. We show the distribution of the coefficient of variation over nodes.
