# Supplementary material for: Controlling target brain regions by optimal selection of input nodes
Source: PLoS Comput Biol. 2024 Jan 12;20(1):e1011274. doi: 10.1371/journal.pcbi.1011274 (PMC10810536; doi:10.1371/journal.pcbi.1011274)
Supplement: S4 Fig — (A) Difference between in-hubs and out-hubs (B) Hubs of the functional connectivity (in-hubs and out-hubs coincide because the functional connectivity is symmetric). Brain regions are colored according to the FC strength (averaged over subjects) (C) Out-hubs of the effective connectivity. Brain regions are colored according to the EC out-strength (averaged over subjects) (D) In-hubs of the effective connectivity. Brain regions are colored according to the EC in-strength (averaged over subjects). Brain images were visualized using BrainNetViewer (Xia M, Wang J, He Y. BrainNet Viewer: a network visualization tool for human brain connectomics. PloS one. 2013;8(7):e68910). (PDF) [file pcbi.1011274.s006.pdf]

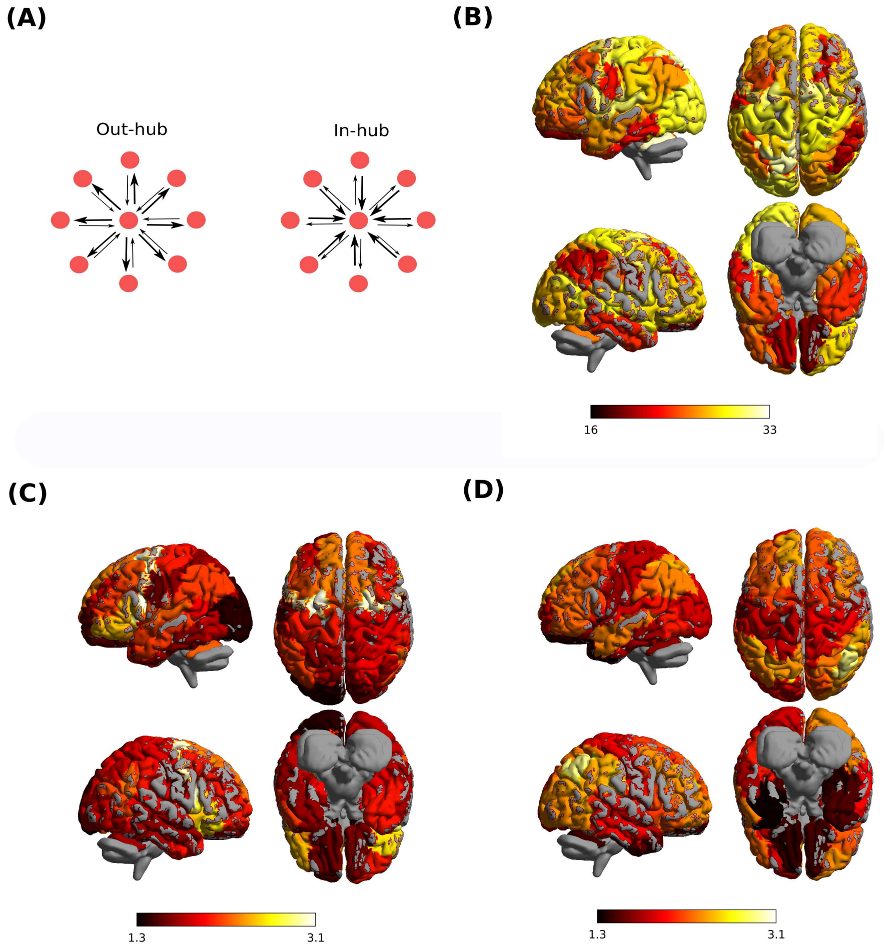

S4 Fig. In-hubs and Out-hubs. **(A)** Difference between in-hubs and out-hubs **(B)** Hubs of the functional connectivity (in-hubs and out-hubs coincide because the functional connectivity is symmetric). Brain regions are colored according to the FC strength (averaged over subjects) **(C)** Out-hubs of the effective connectivity. Brain regions are colored according to the EC out-strength (averaged over subjects) **(D)** In-hubs of the effective connectivity. Brain regions are colored according to the EC in-strength (averaged over subjects). Brain images were visualized using BrainNetViewer (Xia M, Wang J, He Y. BrainNet Viewer: a network visualization tool for human brain connectomics. PloS one. 2013;8(7):e68910).
