# Supplementary material for: Controlling target brain regions by optimal selection of input nodes
Source: PLoS Comput Biol. 2024 Jan 12;20(1):e1011274. doi: 10.1371/journal.pcbi.1011274 (PMC10810536; doi:10.1371/journal.pcbi.1011274)
Supplement: S5 Fig — (A) For each subject, we ranked all nodes based on the value of driver centrality Eid. We show the rank distribution for all nodes, with nodes ordered according to the average rank, from lowest to highest. Nodes are colored according to the resting state network they belong to. (B) Same as (A), but ranks are based on target centrality Eit. (PDF) [file pcbi.1011274.s007.pdf]

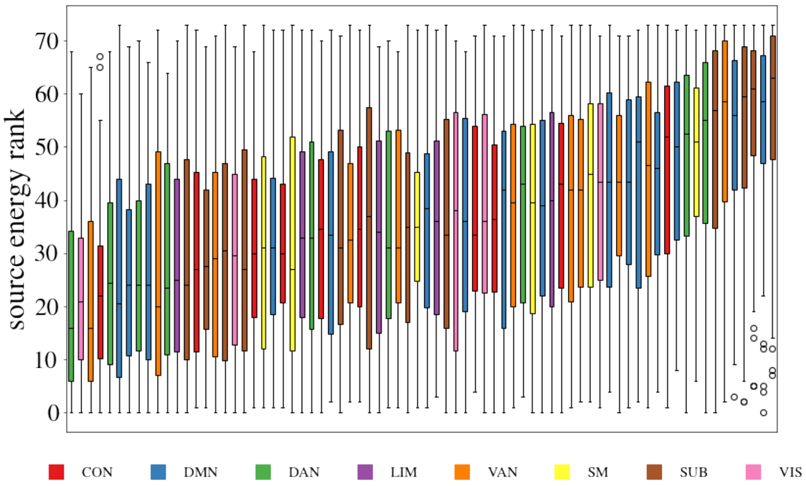

(A) node ranks based on $\mathcal{E}_i^d$

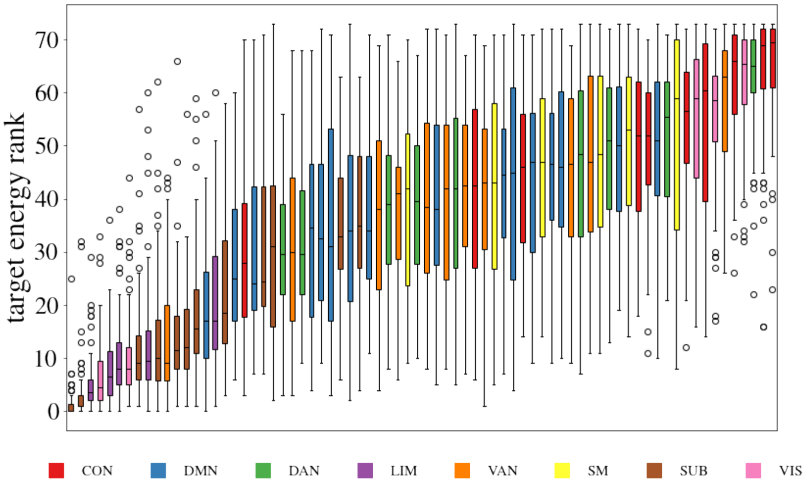

(B) node ranks based on $\mathcal{E}_i^t$

S5 Fig. Node ranks based on driver/target control energy. **(A)** For each subject, we ranked all nodes based on the value of driver centrality $\mathcal{E}_i^d$. We show the rank distribution for all nodes, with nodes ordered according to the average rank, from lowest to highest. Nodes are colored according to the resting state network they belong to. **(B)** Same as (A), but ranks are based on target centrality $\mathcal{E}_i^t$.
