# Supplementary material for: Controlling target brain regions by optimal selection of input nodes
Source: PLoS Comput Biol. 2024 Jan 12;20(1):e1011274. doi: 10.1371/journal.pcbi.1011274 (PMC10810536; doi:10.1371/journal.pcbi.1011274)
Supplement: S6 Fig — Energy to control RSN with a varying number of driver nodes, selecting driver nodes according to the driver centrality (A) Energy to control RSN with a varying number of driver nodes, rescaled by the number of target nodes n. (PDF) [file pcbi.1011274.s008.pdf]

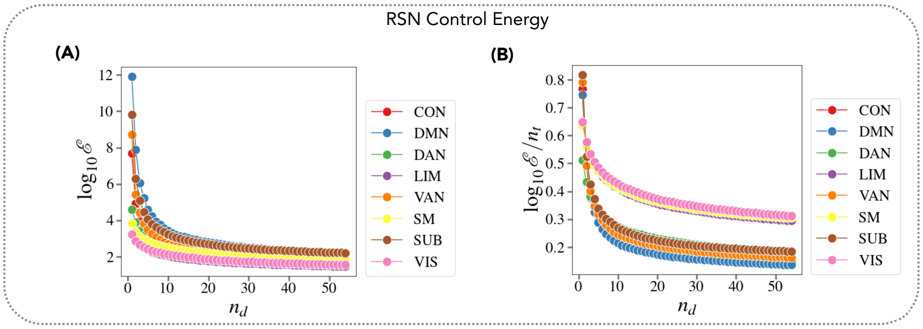

S6 Fig. Dependence of the RSN control energy on the number of driver nodes. **(A)** Energy to control RSN with a varying number of driver nodes, selecting driver nodes according to the driver centrality **(A)** Energy to control RSN with a varying number of driver nodes, rescaled by the number of target nodes n.
