# Supplementary material for: Controlling target brain regions by optimal selection of input nodes
Source: PLoS Comput Biol. 2024 Jan 12;20(1):e1011274. doi: 10.1371/journal.pcbi.1011274 (PMC10810536; doi:10.1371/journal.pcbi.1011274)
Supplement: S7 Fig — For each RSN we show the corresponding target nodes(small blue markers) and top 10 aggregate driver nodes(yellow markers) are shown. Brain images were visualized using BrainNetViewer (Xia M, Wang J, He Y. BrainNet Viewer: a network visualization tool for human brain connectomics. PloS one. 2013;8(7):e68910). (PDF) [file pcbi.1011274.s009.pdf]

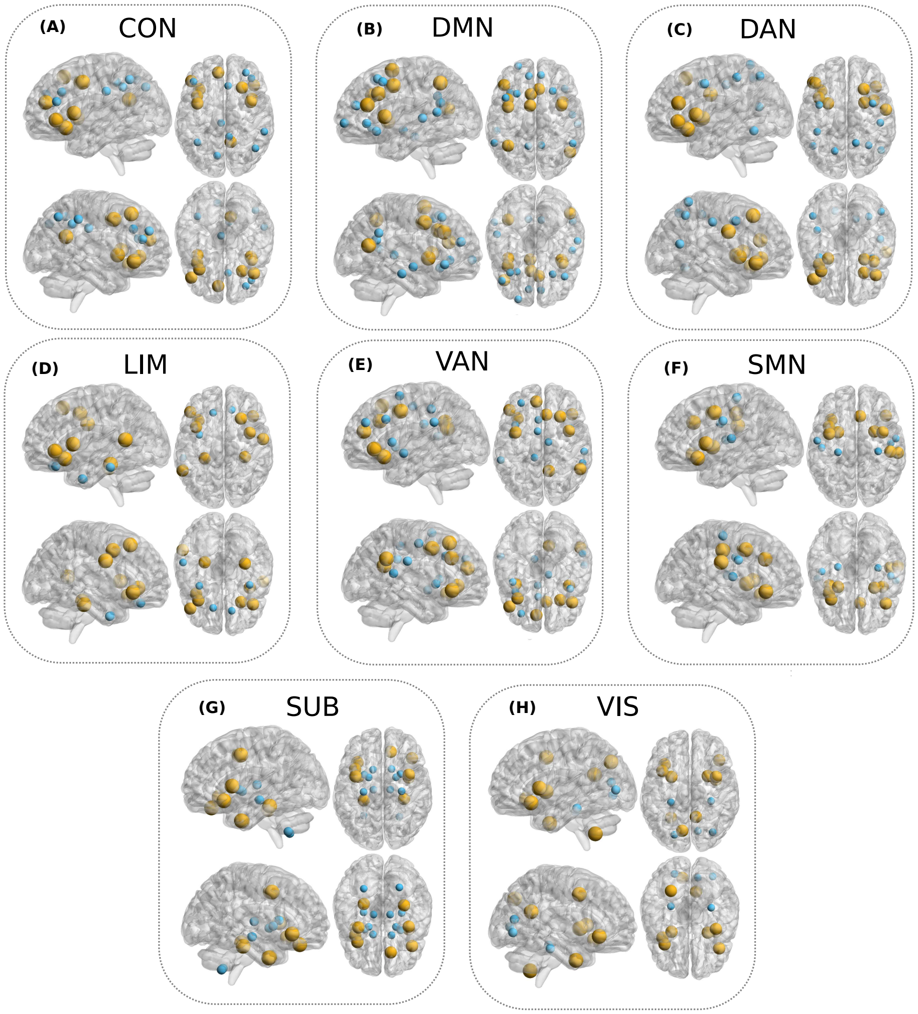

S7 Fig. Optimal driver nodes for RSN. For each RSN we show the corresponding target nodes(small blue markers) and top 10 aggregate driver nodes(yellow markers) are shown. Brain images were visualized using BrainNetViewer (Xia M, Wang J, He Y. BrainNet Viewer: a network visualization tool for human brain connectomics. PloS one. 2013;8(7):e68910).
