# Supplementary material for: Controlling target brain regions by optimal selection of input nodes
Source: PLoS Comput Biol. 2024 Jan 12;20(1):e1011274. doi: 10.1371/journal.pcbi.1011274 (PMC10810536; doi:10.1371/journal.pcbi.1011274)
Supplement: S8 Fig — (A) Energy to control the whole brain network (median over subjects) as a function of the number of driver nodes nd. For each nd, nodes were selected based on a ranking of centrality measures. Nodes had different accessibility values βi in the range [0.01, 1]. (B) Energy to control the whole brain network (distribution over subjects), for three values of nd. For each subject, energy values were z-scored with respect to the mean of the random node selection. (PDF) [file pcbi.1011274.s010.pdf]

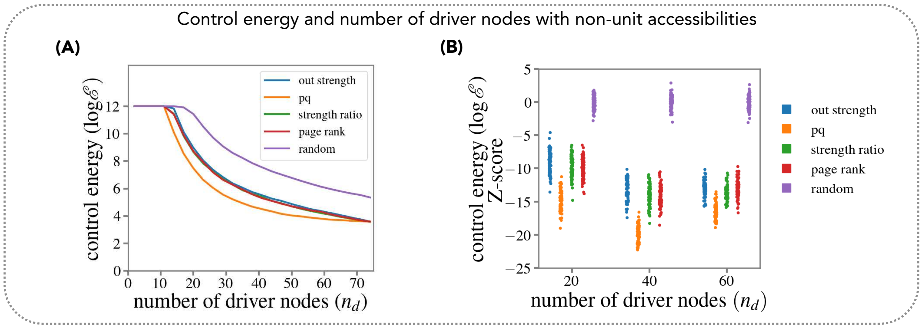

S8 Fig. Control energy in presence of non-unit node accessibilities. **(A)** Energy to control the whole brain network (median over subjects) as a function of the number of driver nodes n_d. For each n_d, nodes were selected based on a ranking of centrality measures. Nodes had different accessibility values β_i in the range $[0.01, 1]$. **(B)** Energy to control the whole brain network (distribution over subjects), for three values of n_d. For each subject, energy values were z-scored with respect to the mean of the random node selection.
