# Supplementary material for: Controlling target brain regions by optimal selection of input nodes
Source: PLoS Comput Biol. 2024 Jan 12;20(1):e1011274. doi: 10.1371/journal.pcbi.1011274 (PMC10810536; doi:10.1371/journal.pcbi.1011274)
Supplement: S9 Fig — (A) Fraction of common nodes (distribution over subjects) among the top-10 ranking nodes according to different centrality measures based on FC and EC. (B) Energy to control target nodes, using nd = 10 driver nodes (distribution over subjects) selected according to different centrality measures. Centrality measures were computed on FC instead of EC. For each number of target nodes, energy values were z-scored with respect to the mean energy obtained with the same centrality but using EC. (PDF) [file pcbi.1011274.s011.pdf]

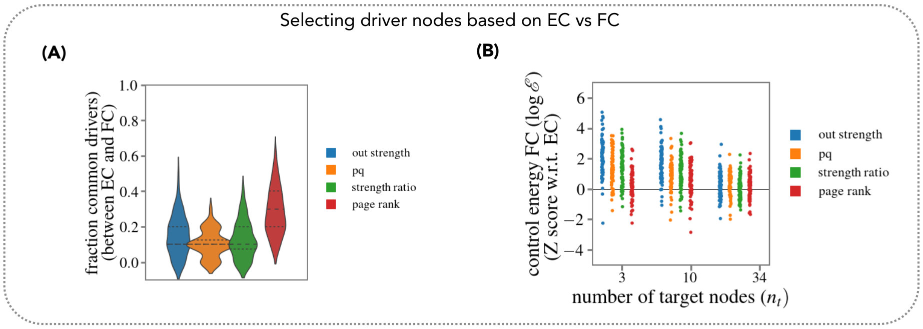

S9 Fig. Selecting nodes based on functional connectivity rather than effective connectivity. **(A)** Fraction of common nodes (distribution over subjects) among the top-10 ranking nodes according to different centrality measures based on FC and EC. **(B)** Energy to control target nodes, using $n_d = 10$ driver nodes (distribution over subjects) selected according to different centrality measures. Centrality measures were computed on FC instead of EC. For each number of target nodes, energy values were z-scored with respect to the mean energy obtained with the same centrality but using EC.
