# Supplementary material for: Controlling target brain regions by optimal selection of input nodes
Source: PLoS Comput Biol. 2024 Jan 12;20(1):e1011274. doi: 10.1371/journal.pcbi.1011274 (PMC10810536; doi:10.1371/journal.pcbi.1011274)
Supplement: S10 Fig — For each subject, we considered the effective connectivity matrix A. We perturbed A by adding random Gaussian noise to each connection. The noise was chosen to have mean 0 and standard deviation proportional to the connection value, i.e., the noise ϵij acting on Aji was ϵij∼N(0,f·Aji) with 0 ≤ f ≤ 0.25. Thus, each link was perturbed by a relative error with magnitude f with f up to 25%. For each value of f, we ranked nodes according to several centrality measures and we assessed the fraction of top-10 ranking nodes common between the unperturbed (f = 0) and perturbed case. (A) Fraction of common nodes (distribution over subjects) among the top-10 ranking nodes according to different centrality measures based on FC and EC. (B) Energy to control target nodes, using nd = 10 driver nodes (distribution over subjects) selected according to different centrality measures. Centrality measures were computed on FC instead of EC. For each number of target nodes, energy values were z-scored with respect to the mean energy obtained with the same centrality but using EC. (PDF) [file pcbi.1011274.s012.pdf]

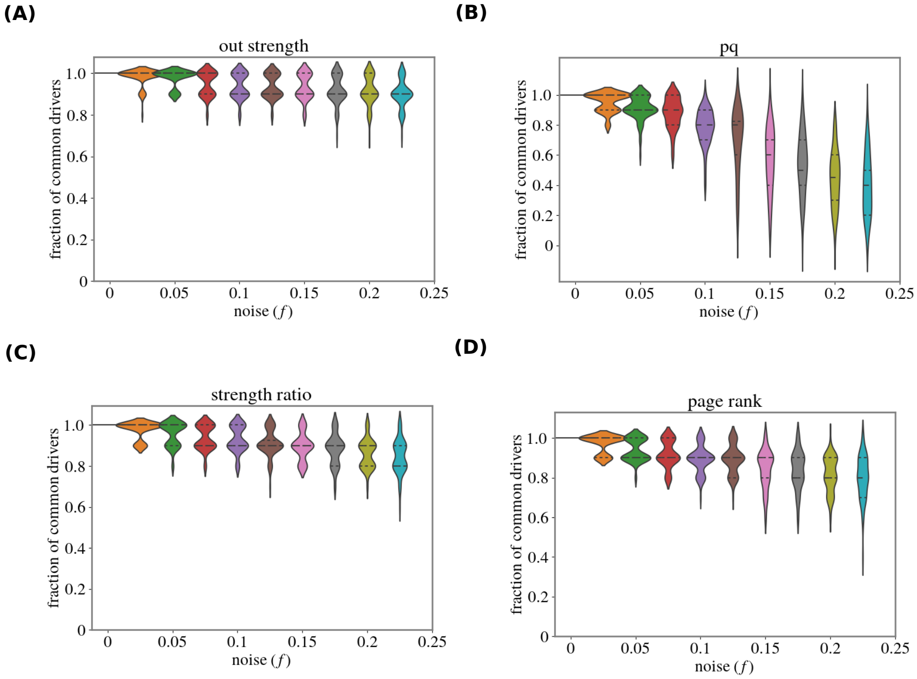

S10 Fig. Effect of perturbing the effective connectivity on driver node selection. For each subject, we considered the effective connectivity matrix A. We perturbed A by adding random Gaussian noise to each connection. The noise was chosen to have mean 0 and standard deviation proportional to the connection value, i.e., the noise ϵ_{ij} acting on A_{ji} was $\epsilon_{ij} \sim \mathcal{N}(0, f \cdot A_{ji})$ with $0 \leq f \leq 0.25$. Thus, each link was perturbed by a relative error with magnitude f with f up to 25%. For each value of f, we ranked nodes according to several centrality measures and we assessed the fraction of top-10 ranking nodes common between the unperturbed ($f = 0$) and perturbed case. **(A)** Fraction of common nodes (distribution over subjects) among the top-10 ranking nodes according to different centrality measures based on FC and EC. **(B)** Energy to control target nodes, using $n_d = 10$ driver nodes (distribution over subjects) selected according to different centrality measures. Centrality measures were computed on FC instead of EC. For each number of target nodes, energy values were z-scored with respect to the mean energy obtained with the same centrality but using EC.
