# Supplementary material for: Controlling target brain regions by optimal selection of input nodes
Source: PLoS Comput Biol. 2024 Jan 12;20(1):e1011274. doi: 10.1371/journal.pcbi.1011274 (PMC10810536; doi:10.1371/journal.pcbi.1011274)
Supplement: S1 Table — We repeated the analysis of control energies for RSN targets, selecting the driver nodes on the basis of centrality measures computed on FC. Here, we show three quantities: i) the average difference in (log-)energies when using group ranking vs individual ranking, based on FC: ΔEFCaggFC=〈log10E〉FC,agg-〈log10E〉FC ii) the average difference in (log-)energies when using individual ranking, based on FC vs EC: ΔEECFC=〈log10E〉FC-〈log10E〉EC iii) the average difference in (log-)energies when using group ranking, based on FC vs EC: ΔEECaggFCagg=〈log10E〉FC,agg-〈log10E〉EC,agg. (PDF) [file pcbi.1011274.s013.pdf]

S1 Table. RSN Target control with functional-connectivity-based node selection. We repeated the analysis of control energies for RSN targets, selecting the driver nodes on the basis of centrality measures computed on FC. Here, we show three quantities: i) the average difference in (log-)energies when using group ranking vs individual ranking, based on FC: $\Delta\mathcal{E}^{FC}_{FC_{agg}} = \langle \log_{10}\mathcal{E}\rangle_{FC,agg} - \langle \log_{10}\mathcal{E}\rangle_{FC}$ ii) the average difference in (log-)energies when using individual ranking, based on FC vs EC: $\Delta\mathcal{E}^{FC}_{EC} = \langle \log_{10}\mathcal{E}\rangle_{FC} - \langle \log_{10}\mathcal{E}\rangle_{EC}$ iii) the average difference in (log-)energies when using group ranking, based on FC vs EC: $\Delta\mathcal{E}^{FC_{agg}}_{EC_{agg}} = \langle \log_{10}\mathcal{E}\rangle_{FC,agg} - \langle \log_{10}\mathcal{E}\rangle_{EC,agg}$.

region	centrality	n_t	n_d	$\Delta\mathcal{E}^{FC}_{FC_{agg}}$	$\Delta\mathcal{E}^{FC}_{EC}$	$\Delta\mathcal{E}^{FC_{agg}}_{EC_{agg}}$
CON	strength ratio	10	10	0.016	0.311	0.237
DMN	page rank	16	10	0.016	0.138	-0.023
DAN	strength ratio	9	10	0.067	0.160	0.152
LIM	page rank	5	10	0.110	0.353	0.358
VAN	page rank	11	10	0.084	0.263	0.152
SMN	page rank	6	10	0.070	0.287	0.205
SUB	page rank	12	10	0.051	0.220	0.097
VIS	page rank	5	10	0.054	0.328	0.233
