# Supplementary material for: Controlling target brain regions by optimal selection of input nodes
Source: PLoS Comput Biol. 2024 Jan 12;20(1):e1011274. doi: 10.1371/journal.pcbi.1011274 (PMC10810536; doi:10.1371/journal.pcbi.1011274)
Supplement: Attachment — Submitted filename: PLOS Reply.pdf [file pcbi.1011274.s014.pdf]

# Response to Reviewer #1

***"This is a valuable contribution to the brain stimulation literature and I have enjoyed reading this manuscript."***

We thank the Reviewer for her/his/their appreciation of our manuscript, accurate reading, useful criticism and insightful comments and suggestions.

"***Some concerns are summarised below***"

In our revised manuscript, we address the concerns expressed by the Reviewer. Changes are detailed here, and visible in blue in the track-change version of the revised manuscript.

"***Depending on the stimulation method chosen (TMS, TACS, DBS etc.), not all nodes would be accessible to drive/control the network. How can we take this into account within this framework? i.e. not all driver nodes, irrespective of centrality, would have the same utility when access is considered. Could the authors incorporate "ability to access a node with certain stimulation techniques" into their centrality measure? (invasive vs noninvasive stimulation techniques)***"

We thank the Reviewer for this appropriate suggestion. In fact, there is a quite natural way to embed this type of accessibility constraint in the framework. The matrix *B* in equation (8) is generally taken to have all nonzero entries equal to 1. This corresponds to assuming that the control signal intensity affects all nodes equally. However, it is absolutely possible to consider non-unit entries in *B*, in particular, $B_{ij} = \beta_i$ where $\beta_i \leq 1$ is the *accessibility* of node *i.* This choice depicts a physical situation where the control signal intensity does not fully reach the node: e.g., $\beta_i = 0.2$ means that the control signal $u\ (t)$ affects node *i* with 20% of its full intensity. Thus, one can naturally incorporate the difficulty of accessing specific nodes in the formalism by modulating the accessibility $\beta_i$ . This modulation obviously affects the entries of the controllability Gramian *W* and the values of control energies.

In this situation, centrality measures defined only on the effective connectivity matrix *A* won't be very effective, as they won't take node accessibility into account. However, consider the pq-centrality (see Methods). The latter is based on the controllability Gramian. The pq-centrality is defined as $r_i = p_i/q_i$

$$p_i = Tr[W^{(i)}] \quad W^{(i)} = \int_0^\infty dt e^{At} b_i b_i^T e^{A^T t} \quad q_i = Tr[M^{(i)}] \quad M^{(i)} = \int_0^\infty dt e^{At} e_i e_i^T e^{A^T t}$$

where $e_i$ is the *i*-th canonical basis vector and $b_i$ is the *i*-th row of *B,* with $b_i = \beta_i e_i$. Thus, we have $p_i = \beta_i^2 Tr[W_0^{(i)}]$ where $W_0^{(i)} = \int_0^\infty dt e^{At} e_i e e^{A^T t}$ is the controllability Gramian obtained in the case where all nodes are fully accessible. In other words, the pq-centrality of

node $i$ scales with node accessibility as $\beta^2_i$, thus accounting for both node topology and node accessibility.

We added a paragraph in the main text (Discussion) and a supplementary figure to discuss node accessibility:

"*Throughout this study, we have assumed that driver nodes can be selected freely. However, depending on the stimulation method chosen (TMS, TACS, DBS etc.), not all nodes might be equally accessible to drive the network. In practice, this means that the control signal cannot reach the nodes, or it can reach it with reduced power. We might define a node accessibility, with* $0 \leq \beta_i \leq 1$, *such that any control signal* $u(t)$ *acting node* $i$ *is reduced by* $\beta_i$ *:* $\beta_i = 1$ *corresponds to fully accessible nodes,* $\beta_i = 0$ *to fully inaccessible ones. This is equivalent to modulating the entries of the matrix* $B$ *in Eq. (8), by taking* $B_{ij} = \beta_i$ *instead of* $B_{ij} = 1$ *for all nonzero entries of* $B$.

*In the presence on non-unit values of the accessibility, centrality measures defined only on the effective connectivity matrix* $A$ *will not lead to a good driver nodes selection (as they do not take node accessibility into account). Conversely, the pq-centrality* $r_i$ *(Methods) is based on the controllability Gramian, which takes into account also the matrix* $B$ *. With non-unit values of the accessibility, we have* $r_i = \beta^2_i r^0_i$, *where* $r^0_i$ *is the value obtained in the case where all nodes are fully accessible. In other words, the pq-centrality of node* $i$ *scales with node accessibility as* $\beta_i$*. Thus, this centrality measure accounts for both node topology and node accessibility.*

*We simulated a situation where nodes had very different accessibilities. We generated random accessibilities between 0.01 and 1, mimicking a situation where some nodes are nearly inaccessible and some are fully accessible. In Supp. Fig. 12 we show the control energy as a function of the number of driver nodes for different driver selection criteria. The lowest energy is obtained using pq-centrality, while the random selection is very inefficient (not surprisingly, as a random selection will generally include low-accessibility nodes).*"

***What does T=10 correspond to in terms of time (i.e. in seconds)? (line 338) Is this a relevant timescale for TMS-EEG studies?***

Times are expressed in units of the repetition time TR=1.4s, so T=10 corresponds to 14s. This is the timescale required for a local perturbation to fully propagate to distal nodes according to the DCM model. We added a figure (Supp. Fig. 8) to show this. We simulated the propagation of a perturbation in the model $\dot{x} = Ax$ where $A$ is the average (over subjects) effective connectivity matrix. The system's state was initialized to x(0) = 0 (corresponding to the stable equilibrium point) except for the state of node $i$ that was perturbed to $x_i(0) = 1$. We let the system evolve freely and observed the functional response of all areas $j \neq i$.

Results are shown in Supp. Fig. 8. The maximum of the functional response is typically in the range $T_{max} \in [5, 30]$ and on average $T_{max} = 10.2$. As we already discussed in Discussion, in real TMS-EEG experiments the functional propagation may be complemented with a more rapid, direct spread of activity along axonal connections.

***While using EC over SC is an interesting approach, are the authors working under the assumption that RSNs are reflective of valid target-driver node definitions for task based TMS-EEG applications? If so, please include relevant experimental evidence as references. I appreciate the authors are somewhat touching upon this as a "limitation: rapid effects of brain stimulation" but I think the issue is not just constrained to propagation of stimulation effects but rather the definition of states that can be modelled as linear and time-invariant over time periods relevant for a stimulation study.***

We acknowledge that our control strategy is primarily intended to be applied in the resting state condition. During rest, we can assume that brain networks remain stable, which enables us to use a time-invariant dynamical model (Eq. 8). In this case, there is evidence that resting state networks (as reconstructed through fMRI) are relevant in terms of driver/target node definition in TMS/EEG experiments. Relevant references are e.g.

Bonnard, M., Chen, S., Gaychet, J., Carrere, M., Woodman, M., Giusiano, B., & Jirsa, V. (2016). Resting state brain dynamics and its transients: a combined TMS-EEG study. *Scientific reports*, *6*(1), 31220.

Ozdemir, R. A., Tadayon, E., Boucher, P., Momi, D., Karakhanyan, K. A., Fox, M. D., ... & Santarnecchi, E. (2020). Individualized perturbation of the human connectome reveals reproducible biomarkers of network dynamics relevant to cognition. *Proceedings of the National Academy of Sciences*, *117*(14), 8115-8125.

Momi, D., Neri, F., Coiro, G., Smeralda, C., Veniero, D., Sprugnoli, G., ... & Santarnecchi, E. (2020). Cognitive enhancement via network-targeted cortico-cortical associative brain stimulation. *Cerebral Cortex*, *30*(3), 1516-1527.

Bonnard et al. (2016) perform a TMS-EEG study where they observe the response of driving nodes from different RSNs at rest. Ozdemir et al. (2021) also perturb different RSN nodes with TMS at rest and observe that responses correlate with behavior in a subsequent task. Momi et al. (2020) that TMS perturbation of different RSN nodes during resting periods enhance subsequent task performance.

However, during tasks, we cannot generally model the dynamics as linear time-invariant (LTI). In fact, the dynamics is commonly modeled as

$$\dot{x}(t) = (A + \sum_i B_i v_i(t))x(t) + Cv(t)$$

where $v_i(t)$ represents (non-controllable) task-dependent inputs that modulate the regions' activity and effective connections (Jung, K., Friston, K. J., Pae, C., Choi, H. H., Tak, S., Choi, Y. K., ... & Park, H. J. (2018). Effective connectivity during working memory and resting states: A DCM study. *NeuroImage*, *169*, 485-495). In this case, the dynamics is non-linear (more specifically bi-linear) and non-time-invariant. There is no general control theory framework to deal with such systems. If the modulation of the effective connectivity is negligible ($B_i = 0$), then the system is linear and one could use control theory for linear non-homogeneous systems (Hampton, R. D., Knospe, C. R., & Townsend, M. A. (1996). A practical solution to the deterministic nonhomogeneous LQR problem); if also the direct node modulation is weak ($C = 0$), then the control problem would again become LTI, and one would only need to replace the rest effective connectivity matrix with a constant task effective connectivity matrix.

To what extent a single task effective connectivity matrix would be a fair approximation, and to what extent the optimal control nodes identified at rest would still be efficient during a task is an open question that would deserve a dedicated study.

We have edited our Discussion session to clarify these points:

"*Task experiments. The dynamical model, Eq. (1), and the EC matrix were derived for the resting state condition. As such, our control model is appropriate during rest. Note that several works already used fMRI-based connectivity results (such as RSNs) to select driver nodes for TMS-EEG experiments [22, 92, 93]. During a task, we cannot generally model the dynamics as linear time-invariant (LTI). In fact, the dynamics can contain task-dependent inputs modulating the regions' activity and effective connections [94], leading to a non-linear and non-time-invariant dynamics. To what extent task-dependent modulations can be neglected, using a single task-related effective connectivity matrix, and to what extent the optimal control nodes identified at rest would still be efficient during a task are an open question that would deserve a dedicated study.* "

***Could you please elaborate further the factors(s) contributing to the asymmetry in the definition of in-hubs vs out-hubs across subjects?***

We have added a supplementary figure (Supp. Fig. 10) to illustrate this point. The in-hubs correspond to nodes with strong incoming connections, out-hubs to nodes with strong outgoing connections (Supp. Fig. 10a). In the effective connectivity matrix, $A_{ji}$ represent a directed link from *i* to *j*, i.e., an outgoing link from *i* and an incoming link to *j.* Since the effective connectivity is non-symmetric, the strength of outgoing and incoming effective connections is not the same. On the other hand, since the functional connectivity is symmetric, the strength of outgoing and incoming functional connections is equal.

In Supp. Fig. 10 (panels b-d) we show a brain surface where brain regions are colored according to the in-strength and out-strength of the effective connectivity, as well as the strength of functional connectivity.

We have correspondingly added a paragraph in the main text.:

"*In Supp. Fig. 10a we illustrate the difference between in-hubs (nodes with strong incoming connections) and out-hubs (nodes with strong outgoing connections) while in Supp. Fig.10 we report brain plots showing the in-strength and out-strength of different regions, and the total FC strength. Our results suggest that "in-hubs'" of effective connectivity are the easiest nodes to control, while "out-hubs"' of the effective connectivity are the best nodes to use to control other nodes, and should possibly be chosen as driver nodes. Intuitively, as the control signal must reach the target node from the driver node, the easiness to control a given target node is related to the existence and the number of network paths leading from the driver to the target. In summary, on average in-hubs are more easily reached (there are many paths leading to them), out-hubs are best drivers (there are many paths leading to the possible targets). Note that in- and out-hubs of EC do not trivially align with FC hubs.*"

***The authors focused primarily on the stimulation-based literature. How does their approach relate to other methods employed in epilepsy surgery: e.g. https://journals.plos.org/ploscompbiol/article?id=10.1371/journal.pcbi.1005637***

In our approach, we identify nodes that should be optimal to transmit external perturbations. Such nodes are not necessarily the nodes that are most relevant for dynamics (e.g., because their removal has a strong impact on the overall dynamics). We added a paragraph to the discussion to clarify this point

"*We stress that the relevance of out-hubs for control, i.e., to transmit external perturbations to distal nodes, does not imply that these nodes are the most relevant or influential in spontaneous brain dynamics. In particular, if one ranks nodes on the basis of the impact their removal} can have on brain dynamics[72], then the most relevant nodes may correspond to other topological criteria, such as belonging to the set of rich-club nodes.*"

***Minor comments: the authors should carefully review their spelling throughout the manuscript – an example sentence 667-672 however this issue is present throughout.***

We have carefully polished the manuscript.

## Response to Reviewer #2

***"Manjunatha et al. present a manuscript introducing a novel methodology to estimate brain "controllability" from (asymmetric) estimates of effective connectivity (EC) estimated using sparse Dynamic Causal Modelling (sDCM). Using these directed graphs (fit to subject level resting state fMRI), the authors deploy Kalman's controllability framework to identify optimal nodes as either "drivers" or "targets" as determined by a theoretical energy requirement dependent on graph properties alone. The authors provide convincing evidence that identified "in" and "out" hubs in the brain have distinct energy demands for control. The paper is precise and well written, and present a number of insightful findings."***

We thank the Reviewer for her/his/their appreciation of our manuscript, accurate reading, useful criticism and insightful comments and suggestions. In our revised manuscript, we address the concerns expressed by the Reviewer. Changes are detailed here, and visible in blue in the track-change version of the revised manuscript.

***"The paper is highly theoretical however, and lacks any analysis of sensitivity or validation of the method that would improve its impact. I have a few issues that I recommend be addressed:"***

The main goal of our manuscript is to propose a theoretical framework for brain control. A true validation of our framework can only occur experimentally, by testing its predictions in actual neurostimulation experiments. However, we agree with the Reviewer that we should better explain the potential gains of our method, and its robustness. We have added several control analyses aimed at clarifying these points.

"***This paper introduces EC estimates to replace more standardly used functional***

***connectivity estimates of graph connectivity. I understand that asymmetry allows for hubs to be better characterized, but it is not clear exactly what is lost/gained. For instance, what are the overlaps in nodes identified with FC vs EC approaches? I suspect also that the EC estimates introduces much more variability between subjects, so how does EC and FC compare when performing group level identification of optimal nodes.***"

We agree that this is a relevant point. To clarify this issue, we repeated the analysis selecting driver nodes on the basis of centralities computed on FC (instead of EC). Results are reported in the new Supp. Fig. 15 and Table 2.

The high-ranking nodes (e.g., the top 10 nodes) identified on the basis of EC or FC significantly differ. On average, there is an overlap of 10-20%, depending on the centrality measure (Supp. Fig. 15a). In fact, the in- and out- hubs of EC do not simply align with FC hubs. This is apparent in the new Supp. Fig. 10, where we show a brain surface where brain regions are colored according to the in-strength and out-strength of the effective connectivity, as well as the strength of functional connectivity.

In general, selecting driver nodes on the basis of EC affords an advantage in terms of control energy. In Supp. Fig. 15b we show the average control energies to control a certain number of target nodes, with driver nodes selected according to centrality measures computed on FC. Values have been Z-scored by subtracting the mean and dividing by the standard deviation of the corresponding values obtained with EC-based selection of driver nodes. For a small number of target nodes, the FC-based selection of driver nodes consistently gives larger values of control energy (remember that for a large number of target nodes, $n > 20$ , there is no advantage altogether of using centrality measures over randomly selecting driver nodes.

We added a paragraph in Results: Scaling with the number of target nodes to report this analysis:

"*We stress that centrality measures are computed directly on the EC, not on standard FC. In Supp. Fig. 15a we show the overlap between the 10 highest-ranking nodes selected with the EC and with the FC, respectively. On average (over subjects),the overlap was only 15% (for out strength), 11% (for pq-centality), 13% (for degree ratio centrality), 28% (for page rank centrality). Correspondingly, in Supp. Fig. 15b we show the distribution of the (log-)control energies obtained using FC-based instead of EC-based centrality measures. The values of the (log-)control energy were z-scored to the mean of the distribution obtained with an EC-based choice of driver nodes. The FC-based choice consistently yields larger energies than the EC-based one.*"

We also repeated the analysis of control energies for RSN targets, selecting the driver nodes on the basis of centrality measures computed on FC. In particular, we repeated the comparison of control energy with individual vs. group selection of driver nodes (as in Table 1). Since FC estimates are less variable over subjects, one might expect a smaller difference when comparing individual and group selection. Results are reported in Table 2. Here, we show three quantities:

- the average difference in (log-)energies when using group ranking vs individual ranking, based on FC: $\Delta E_{FC}^{FC_{agg}} = <\log_{10}E>_{FC_{agg}} - <\log_{10}E>_{FC}$ . Consistent with expectation, the difference is generally smaller than the difference obtained using EC. Indeed, on average over RSNs we have $\Delta E_{FC}^{FC_{agg}} = 0.059$ compared with $\Delta E_{EC}^{EC_{agg}} = 0.152$ obtained with EC.
- ii) the average difference in (log-)energies when using individual ranking, based on FC vs EC: $\Delta E_{EC}^{FC} = <\log_{10}E>_{FC} - <\log_{10}E>_{EC}$ . The difference is always positive. On average over RSNs we have $\Delta E_{EC}^{FC} = 0.257$.
- iii) the average difference in (log-)energies when using group ranking, based on FC vs EC: $\Delta E_{EC_{agg}}^{FC_{agg}} = <\log_{10}E>_{FC_{agg}} - <\log_{10}E>_{EC_{agg}}$ . The difference is always positive, except for DMN (where the $\Delta E_{EC_{agg}}^{FC_{agg}} =- 0.024$). On average over RSNs we have $\Delta E_{EC}^{FC} = 0.180$.

Thus, while using FC to select centrality measures allows reducing the “energy gap” obtained when using group selection instead of average selection, control energies are still larger than those obtained when using EC-based centrality measures.

We added a paragraph in Results: Individual vs. group selection of driver nodes:

“*We also verified that selecting driver nodes based on centrality measures computed on the FC (instead of the EC) leads to larger control energies. Results are shown in Table2, where we compare three quantities: i) the average difference in (log-)energies when using group ranking vs individual ranking, based on FC. Since FC estimates are less variable over subjects than EC estimates, the difference is generally smaller than the difference obtained using EC ii) the average difference in (log-)energies when using individual ranking, based on FC vs EC. The difference is always positive iii) the average difference in (log-)energies when using group ranking, based on FC vs EC. The difference is always positive, except for DMN. Thus, while using FC to select centrality measures allows a greater robustness of group results, control energies are still larger than those obtained when using EC-based centrality measures. Notably, when using FC, the optimal centrality criterion is nearly always the page rank centrality, implying that optimal control nodes are generally those that \textit{do not} receive many incoming connections (hence, not FC hubs).*”

“***How sensitive is the controllability analysis and following identification of node optimality affected by small variations in EC estimates? If we prune away or invert a particular connection, how much does node selection change? A perturbation***

***analysis, taking into account the posterior precisions over connectivity parameters would be particularly interesting to this respect.***"

We agree with the reviewer's comment that we should demonstrate the robustness of our results with respect to variations in EC estimates. We performed a perturbation analysis by adding random Gaussian noise to each connection in the effective connectivity matrix A. The noise was chosen to have mean 0 and standard deviation proportional to the connection value, i.e., the noise $\varepsilon_{ij}$ acting on $A_{ij}$ was $\varepsilon_{ij} \sim N(0, f \cdot A_{ij})$ with $0 \leq f \leq 0.25$. Thus, each link was perturbed with a relative error of up to 25%.

For each value of the noise strength *f,* we ranked nodes according to several centrality measures, and we assessed the fraction of top-10 ranking nodes common between the unperturbed and perturbed case. Results are now shown in Supp. Fig. 16. The set of optimal nodes is very robust with respect to small variations of the effective connectivity estimates. On average (over subjects) the fraction of top-ranking nodes stays above 90% (for out strength centrality), above 85% (for ratio degree centrality), above 40% (for pq centrality) for values of noise up to 25%. Note that the pq centrality is more fragile to noise, because adding gaussian noise has the effect of perturbing the spectral properties of *A*, creating large eigenvalues (which should not occur), which reflect into the spectral properties of the Gramian.

We added a paragraph in Discussion: Effective-connectivity-based controllability to discuss this point:

"*To ensure that our EC-based scheme is not sensitive to small errors in EC estimates, we performed a sensitivity analysis. For each subject independently, we added random Gaussian noise in such a way as to perturb each effective connection with a relative error f of up to 25%. For each value of f, we assessed the fraction of top-10 ranking nodes (according to several centrality measures) common between the unperturbed and perturbed case. Results are shown in supplementary Fig. 16. The set of optimal nodes is very robust with respect to small variations of the effective connectivity estimates. On average (over subjects) the fraction of top-ranking nodes stays above 90% (for out strength centrality), above 85% (for ratio degree centrality), above 40% (for pq centrality) for values of noise up to 25% (the pq centrality is more fragile, because adding Gaussian noise has the effect of perturbing the spectral properties of A, creating large eigenvalues which strongly distort the spectral properties of the Gramian).*"

***The paper would be more impactful if it contained an empirical validation to test the underlying assumptions of the approach. The introduction states current failures of controllability arise from "the usage of inaccurate computational models of brain dynamics". This work deploys directed connectivity as its principle novelty – does this added description suffice to improve controllability? A set of numerical simulations of delayed, nonlinear models could help validate the approach by demonstrating that simplifying assumptions (such as ignoring node-dynamics or delays) can still yield prediction useful in real world systems. I understand this might be out the scope of current paper, so leave it to the authors as to how best to address***

***this.***

The term ‘accurate’ we employed was perhaps not the best in this context (also because ‘accurate’, in modeling jargon, is sometimes used to refer to an unbiased estimate of biophysical parameters). We thus modified the introduction as follows:

“*A second relevant obstacle is the choice of the model for brain dynamics… In the present work, we propose a controllability approach relying on a realistic control objective and an EC-based dynamical model able to well fit the observed fMRI data.”*

The DCM we used is a sound model of brain dynamics, as all parameters have a clear biophysical interpretation, and the model fit the data very nicely (e.g., achieving a Pearson correlation between the empirical and simulated time series of 0.9). However, its main limit is that it involves a first-order approximation (which is why it is linear). We agree with the Reviewer that directed connectivity, the key feature of our DCM model, may not be the only relevant factor for controllability, and testing our approach on more complex, nonlinear, whole-brain models would be an important validation step. In this sense, the best idea would be to test our predictions on a model of intermediate complexity, retaining directed connectivity but also adding some dynamical complexity. A good candidate model would be the model proposed by [Deco et al., *PNAS*, *116*(36), 18088-18097], which includes non-linear oscillators coupled by directed effective connections. We think that such an analysis falls outside the scope of this paper and would be best addressed in a follow-up study.

We added a paragraph in Discussion: limitations to mention this point:

*“Whole-brain computational model. The DCM model used in this study can yield a very accurate estimate of the effective connectivity, but is dynamically simplistic. To what extent predictions of our linear model would be fully accurate in the case of a more complex, non-linear node dynamics is an open question. In future work, we will address this question by testing our predictions on simulations of several whole-brain models with non-trivial node dynamics. To this aim, we will use models including a data-driven, asymmetric EC estimate (the key feature for our approach) and non-trivial node dynamics, such as the model proposed by Deco et al. [95].”*

“***To make accessible to more general audience – could you address how this stimulation approach would look in practice?***”

We edited the introduction to better clarify this point:

“*The approach we propose has the potential to inform neurostimulation experiments with non-invasive techniques such as transcranial magnetic stimulation or temporal interference ([30,31]). These techniques could be used to control the activity of a (small) set of target regions, by inducing a desired activity pattern (i.e., activating or de-activating specific regions). To this aim, given estimates of each subject's effective connectivity obtained with fMRI, our approach allows identifying the optimal driver region (or set of driver regions) and*

*assess the control energy (the amplitude of the control signal to be applied) necessary to control the target.*"

"***The algorithm has been further adjusted to account for the signal reliability of the temporal frames, by introducing the binary temporal mask as a weighting measure during the estimation procedure." – its not clear what this means, could you please clarify?***"

We agree that this point was not clear. We replaced the sentence:

"*high-motion frames (with framewise displacement FD>0.4) were weighted less than normal frames (FD<0.4) in the estimation algorithm*."

***L201 - a textual description of the interpretation of driver and target centrality metrics follows later in the text, I think it would be clearer to move this up to this point.***

A description of the interpretation is now given when introducing the two centralities:

"*The driver energy represents the average energy with which we can control another node, using node i as a driver. The target energy represents the average energy with which we can control node i using another node as a driver.*"

*"**Figures –axis labels use mathematical notation which makes them hard to read outside of the text. I would suggest to use natural language and be clear to indicate which colour legends apply to which figures.***"

We edited figures according to the Reviewer's suggestion. Now axes labels present natural language and color legends can be unambiguously matched to the relevant panels.

***The intuition behind the explanation underlying the identification of in-hubs as optimal targets for stimulation. I would also expect that an element of controllability pertains to how much "competition" a node is receiving. If the driving node is competing with lots of other extraneous nodes for control then surely the energy will be higher?***

As a control signal will spread from the driver node to many downstream nodes, any target node will "compete" with all other nodes for controllability. *On average,* in-hubs are easier target nodes, because there are many paths from the driver node to these targets. However, the larger the number of nodes, the larger the number of nodes with which the target node is competing: indeed, the control energy is larger when the number of nodes is larger.

We have added some explanation to clarify this point:

"*Our results suggest that "in-hubs" of the effective connectivity are the easiest nodes to control, while "out-hubs" of effective connectivity are the best nodes to use to control other nodes, and should possibly be chosen as driver nodes. Intuitively, as the control signal must*

*reach the target node from the driver node, the easiness to control a given target node is related to existence and number of network paths leading from the driver to the target: thus, on average in-hubs are more easily reached (there are many paths leading to them), out-hubs are best drivers (there are many paths leading to the possible targets). Note that in- and out-hubs of EC do not trivially align with FC hubs.*"

***"Figure 3 – I am not sure what these are meant to show. That good drivers are more frontally distributed? At the moment this isn't entirely clear.***"

The figure was meant to show where good driver/target nodes are located in the brain. However, we think that the brain representation we used was not suitable to clearly convey this information. We replaced the previous figure with a new figure where color code directly related to input/target control energy. This new figure should be much clearer.

***Nodes with low rank L391 – should this be "high rank"?***

We have edited the text and figures to consistently use the terms "low rank"/"high rank". Nodes are ranked according to the centrality values in increasing order, so that "good nodes" are "high-ranking nodes".

***Can you normalize the energy by the RSN size? You've already shown smaller networks are going to be easier to control, so a normalized metric would be useful.***

We followed the Reviewer's suggestion. We thus added a new panel (Supp. Fig. 12a) and some text to discuss the normalized metric:

"*In Supp. Fig. 12b we show the average (log-)energy, normalized by the number of target nodes included in each network. This allows a more direct comparison between different RSNs. It is clear that the DMN, DAN, CON, VAN and SUB are comparatively easier to control, while LIM, VIS and SMN are more difficult to control.*"

***Figure 5a – what is the reasons for doing these analyses? Do you hypothesize that one RSN drives another? This is not really considered in the introduction.***

We better clarified this in the introduction:

"*Then, we will consider the case where one aims to control interconnected groups of regions defined by canonical resting state networks (RSNs). Controlling RSNs could be of clinical importance, as several brain diseases perturb specific RSNs. This analysis will also allow us to assess whether specific RSNs can be preferentially controlled from other RSNs, which could be of potential relevance in discussions of cognitive control*"

***What are the biases inherent in estimating the EC? Do regions with a stronger BOLD signal tend to be demarked as in- or out- hubs? Can this explain why out-hubs have***

***high variability, because they tend to be located in regions of low SNR?***

Nodes with larger BOLD signal (st. dev.) tend to be demarcated as in-hubs (the correlation between node standard deviation and in-strength is R=0.47). Also see below a map of node standard deviation and compare with the in-strength map in Supp. Fig. 10d showing in-hubs. Note that this need not be a bias, it can well be a meaningful biological relation (e.g., nodes receiving input from many other nodes have larger signals). There is very weak (positive) correlation (R=0.12) between node standard deviation and out-strength. Thus, the SNR cannot easily explain why out-hubs are variable across subjects.

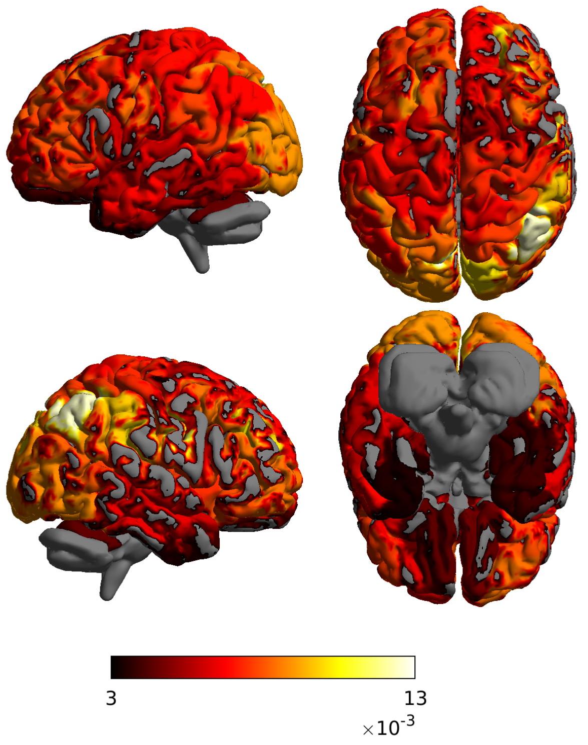


***I suspect that the discussion relating to "optimal driver regions" is going to change depending on what type of data you have (i.e., resting or task based). Could you comment on how stable these driver optimalities are likely to be?***

Our framework is primarily intended for the resting condition. We have added a paragraph in Discussion: limitations to discuss its possible extension to task data:

"*Task experiments. The dynamical model, Eq. (1), and the EC matrix were derived for the resting state condition. As such, our control model is appropriate during rest. Note that several works already used fMRI-based connectivity results (such as RSNs) to select driver nodes for TMS-EEG experiments [22, 92, 93]. During a task, we cannot generally model the dynamics as linear time-invariant (LTI). In fact, the dynamics can contain task-dependent inputs modulating the regions' activity and effective connections [94], leading to a non-linear and non-time-invariant dynamics. To what extent task-dependent modulations can be neglected, using a single task-related effective connectivity matrix, and to what extent the*

*optimal control nodes identified at rest would still be efficient during a task are open questions that would deserve a dedicated study.*"
